# Recovery of Tantalum and Manganese from Epoxy-Coated Solid Electrolyte Tantalum Capacitors through Selective Leaching and Chlorination Processes

**DOI:** 10.3390/ma15020656

**Published:** 2022-01-16

**Authors:** Wei-Sheng Chen, Chih-Yuan Hsiao, Cheng-Han Lee

**Affiliations:** Department of Resources Engineering, National Cheng Kung University, No. 1 Daxue Road, Tainan 701401, Taiwan; kenchen@mail.ncku.edu.tw

**Keywords:** tantalum capacitor, manganese, selective leaching, chlorination, recovery

## Abstract

Electronic products are ever growing in popularity, and tantalum capacitors are heavily used in small electronic products. Spent epoxy-coated solid electrolyte tantalum capacitors, containing about 22 wt.% of tantalum and 8 wt.% of manganese, were treated with selective leaching by hydrochloric acid and chlorination after removing the epoxy resin, and the products converted, respectively, to Mn(OH)_2_ and TaCl_5_. The effects of acid type, acid concentration, liquid–solid ratio, and reaction time were investigated to dissolve the manganese. The optimal selective leaching conditions were determined as 3 mol/L of HCl, 40 mL/g at 25 °C for 32 min. Next, residues of selective leaching after washing and drying were heated with ferrous chloride to convert to pure TaCl_5_. Mixing 48 wt.% of chloride and 52 wt.% of residues for a total of 5 g was conducted to complete the chlorination process in the tube furnace at 450 °C for 3 h. A total of 2.35 g of Ta was collected and the recovery of Ta achieved 94%. Finally, Mn(OH)_2_ and TaCl_5_ were separated and purified as the products.

## 1. Introduction

The demand for tantalum capacitors is steadily increasing due to the popularity of small electronic products. At the same time, small electronic products are eliminated rapidly, which represents a large amount of waste [1,2,3]. Tantalum is a transition element with an atomic number of 73, an atomic weight of 180.95, and a melting point of about 3000 °C (2980 ± 20 °C), which is slightly lower than tungsten and rhenium. The resources of tantalum, which usually co-exist with niobium, are mainly from the coltan or columbite [4,5], making refinement difficult due to their similar chemical characteristics. Tantalum was discovered in 1802, one year after niobium, and the average amount of tantalum in the Earth’s crust is 2 ppm [6]. Therefore, it is feasible to recover tantalum from tantalum capacitors that do not contain niobium [7,8,9]. Tantalum has high capacitance/unit volume, high thermal stability, and high oxidation resistance which can be used in electronic and biotechnology industries [10,11,12]. In the year 2016, about 34% of tantalum was used in manufacturing capacitors, followed by superalloy, chemical, sputtering target, mill product, and carbide industries [11,13].

Epoxy-coated solid electrolyte tantalum capacitors (EcSETCs) consist of electrodes, an epoxy resin, and wires. Electrodes include cathodes, anodes, and dielectrics. Anodes and dielectrics are made of tantalum and a small amount of its oxide powder, while cathodes are made of manganese oxide, graphite, and silver paste. The epoxy resin, which has silicon added to enhance its thermal durability, comprises halogenated compounds.

Tantalum-rich electrodes are sealed with epoxy resin. Hence, the removal of epoxy resin is conducted before any recovery process. Many methods can remove the epoxy resin, such as combustion [14], pyrolysis [15,16,17,18], solubilization [19,20], and supercritical water treatment [21]. After removing the epoxy resin, the recovery of tantalum mainly chooses one of two methods: the hydrometallurgy process and the chlorination process. In the hydrometallurgy process, leaching agents such sulfuric acid (H_2_SO_4_) [22,23], hydrofluoric acid (HF) [24], or a mixture of both [14,15], all applied at normal temperatures, have been investigated. Previous studies have indicated that pressure leaching using HF has better leaching efficiency [7]. After leaching, several extractants such as MIBK [25], CHO [26], and Alamine 336 [17,27] have been studied. The drawbacks of traditional extraction are high volatility and low thermal stability. To overcome these shortcomings, ionic liquids are studied in hydrometallurgy [24,28,29]. In the chlorination process, chlorine gas or hydrogen applied to tantalum and niobium raw ore [6] or ferrous chloride applied to tantalum capacitors [15,30,31,32] have both been studied.

In most of the studies mentioned above, the recovery of tantalum is higher in the hydrometallurgy process but the purify of tantalum is higher in the chlorination process. In this study, a feasible recycling process that combines the advantage of hydrometallurgy and chloride metallurgy was designed. After first removing the epoxy resin from EcSETCs, selective leaching was conducted to remove the manganese and increase the recovery of tantalum. Previous studies on leaching manganese from the different wastes were researched [33,34,35,36,37,38] to investigate the best leaching efficiency and increase the recovery rate of manganese. Low concentrate ordinary inorganic acid, which is applied to dissolve the manganese through selective leaching, was chosen instead of hydrofluoric acid, which is more dangerous and a pollutant. The parameters were investigated, such as acid concentrate, liquid–solid ratio, and reaction time to increase the leaching efficiency of manganese. Then, manganese hydroxide was obtained through chemical precipitation. Furthermore, chlorination with ferrous chloride was used in the recovery of tantalum. The manganese hydroxide and the tantalum chloride were separated individually as the final productions in this study.

## 2. Materials and Methods

### 2.1. Materials

The EcSETCs in this study were made in Taiwan with 100 µF/16 Volt. An EcSETC include an epoxy resin, wires, and an electrode. The mass ratio of each physical component is shown in Table 1. The elements in EcSETCs were dissolved with 90% *v*/*v* of aqua regia and 10% *v*/*v* of hydrofluoric acid and the ion concentration in the solution measured by inductively coupled plasma optical emission spectrometry (ICP-OES; VISTA-MPX, Varian, Palo Alto, CA, USA). The concentration of the main element compositions is shown in Table 2. The sulfuric acid (≥98.0%), nitric acid (≥65.0%), hydrofluoric acid (≥45%), hydrochloric acid (≥37.0%), and ferrous chloride (≥99%) were purchased from Uni-Onward Corp (New Taipei City, Taiwan). Chemicals used in this study were all of the analytical quality. The purity of the nitrogen gas used in pyrolysis was 99.0% from Yunshan Gas Co., Ltd. (Tainan, Taiwan).

### 2.2. Experimental Procedures

#### 2.2.1. Pretreatment

Silicon added in the epoxy resin is the main factor affecting tantalum recycling because it is recovered with tantalum through chlorination. On the other hand, iron and nickel from wires also affect the efficiency of selective leaching. Due to the reasons mentioned above, pretreatment is necessary. The epoxy resin can be removed through pyrolysis with nitrogen gas. Thermogravimetric analysis (TGA, Perkin Elmer, Pyris Diamond TG/DTA, Waltham, MA, USA) was used to determine the pyrolysis temperature in the pretreatment. After the pyrolysis process, grinding and washing with deionized water to flush the epoxy resin was conducted. Next, the electrode was crushed to increase the specific surface area that can improve the efficiency of selective leaching. Finally, the iron and nickel were removed from the wires through magnetic separation. The results of samples without epoxy resin and wires after pretreatment were analyzed by the multi-function environmental field emission scanning electron microscope (EFE-SEM; SU-5000, HITACHI, Chiyoda ku, Japan) with electronic data systems (EDS; EDAX, Mahwah, NJ, USA) and ICP-OES.

#### 2.2.2. Selective Leaching

After the pretreatment, the samples were dissolved by the three kinds of inorganic acids. Hydrochloric acid, nitric acid, and sulfuric acid were chosen. The leaching agent with the best efficiency of selective leaching was chosen. Next, parameters such as acid concentration, liquid–solid ratio (L/S ratio), and reaction time were studied. The effect of acid concentration from 0.25 M (mol/L) to 5 M (mol/L), L/S ratio from 10 mL/g to 50 mL/g, and reaction time from 2 min to 256 min were set to increase the efficiency of selective leaching in this study. After the best parameters were tested and chosen, precipitating manganese hydroxide through adjusting the pH value was conducted. Next, manganese hydroxide was obtained through calcination as one of the final productions in this study.

#### 2.2.3. Chlorination

**Figure 1:**
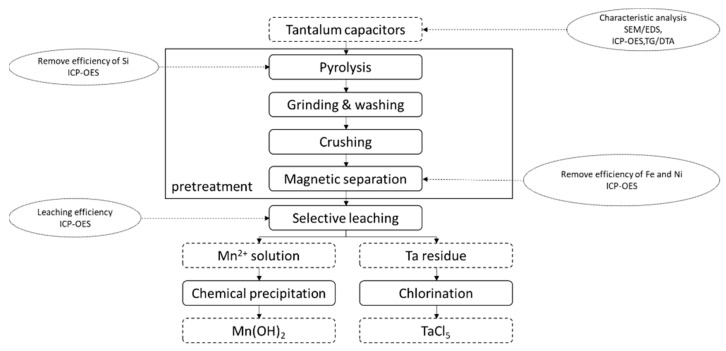
The recovery process of tantalum from EcSETCs. SEM/EDS: multi-function environmental field emission scanning electron microscope with electronic data systems. ICP-OES: inductively coupled plasma optical emission spectrometry. TG/DTA: Thermogravimetric analysis.

The residues from the selective leaching process were washed and dried. Subsequently, the samples were heated in the tube furnace with ferrous chloride. After the stoichiometry, 48 wt.% of residues of selective leaching and 52 wt.% of ferrous chloride were chosen. Tantalum was collected in the form of TaCl_5_. The experiment process is shown in Figure 1.

## 3. Results and Discussions

### 3.1. Pretreatment

The epoxy resin and wires are the main factors to affect the recovery efficiency. Therefore, removing the epoxy resin and wires is the first step before any recovery process. A pretreatment process was conducted in this experiment.

**Figure 2:**
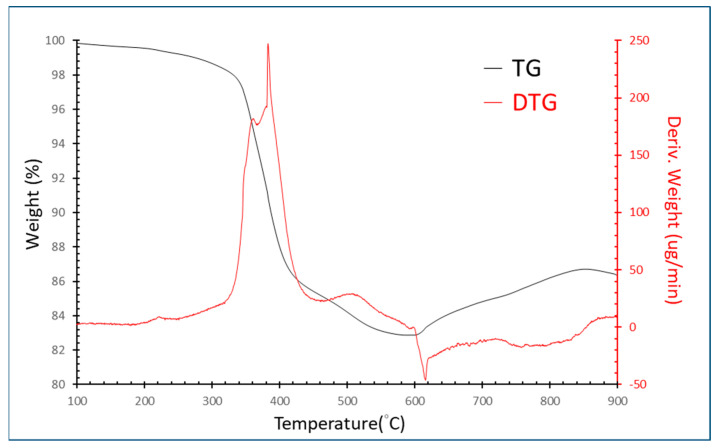
TG/DTG curves of EcSETCs with nitrogen gas (N_2_).

**Figure 3:**
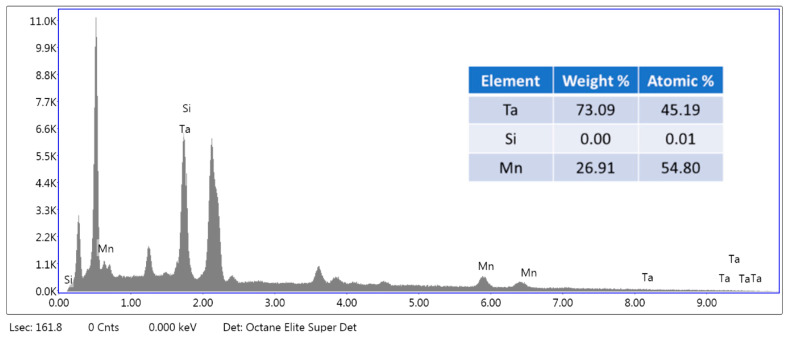
The SEM-EDS patterns of the samples after pyrolysis, grinding, washing, and crushing and the quantitative analysis of samples by SEM-EDS.

Figure 2 illustrates the TG/DTG patterns showing that the first decomposition stage began at 350 °C until 420 °C. Then, the second stage of decomposition is from 420 °C to 600 °C. Due to the two stages of decomposition, we speculate that the epoxy resin has two mainly organic phases. According to the TG/DTA patterns, 600 °C was chosen, and the holding time found through experiments was 10 min to avoid burning the electrodes. After the pyrolysis process, grinding and washing were conducted to remove the epoxy resin. The SEM-EDS patterns are shown in Figure 3, which demonstrates that silicon was removed from the samples. Next, the wires with iron and nickel were removed through a magnetic separation after crushing. Table 3 shows the removed efficiency of silicon, iron, and nickel in the samples after pretreatment. These data showed that silicon, iron, and nickel were successfully removed through pretreatment. 

### 3.2. Selective Leaching for Manganese Separation

After pretreatment, three kinds of inorganic acids were chosen to discuss manganese leaching efficiency. As tantalum does not dissolve in the typical inorganic acids, Table 4 shows that the leaching efficiencies of tantalum are quite low in three kinds of inorganic acids. On the other hand, Table 4 shows that hydrochloric acid had the best leaching efficiency of manganese. Therefore, this study chose hydrochloric acid as a leaching agent to selective leach from the samples after pretreatment.

#### 3.2.1. Effect of the Hydrochloric Acid Concentration

**Figure 4:**
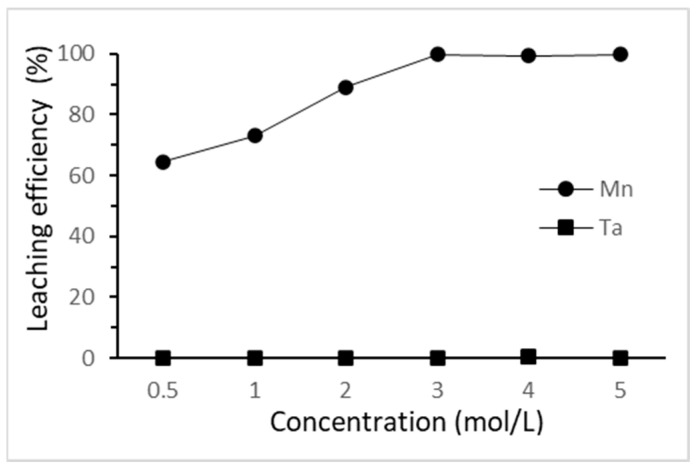
Effect of HCl concentration on the selective leaching efficiency. (L/S ratio: 50 mL/g; reaction time: 3 h; reaction temperature: 25 °C).

The effect of HCl concentration on the selective leaching of tantalum and manganese was investigated from 0.5 M to 5 M at 25 °C for 3 h. The results are shown in Figure 4. The leaching efficiency of manganese increased as the hydrochloric acid concentration increased from 0.5 M to 3 M. The fact that tantalum did not dissolve through this step was confirmed. The highest leaching efficiency of manganese was achieved when hydrochloric acid concentration reached 3 M. On the other hand, the minimal amount of silver in the cathodes was not dissolved due to the chloride ion from the hydrochloric acid. Here, 3 M of hydrochloric acid was chosen as the optimal concentration in this study.

#### 3.2.2. Effect of the Liquid–Solid Ratio

**Figure 5:**
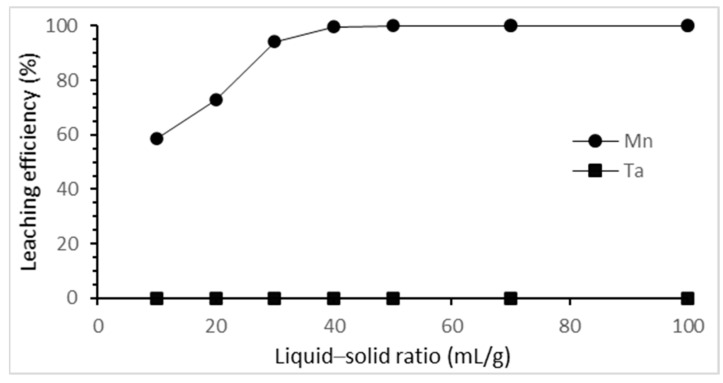
Effect of liquid–solid ratio on the selective leaching efficiency. (HCl concentration: 3 M; reaction time: 3 h; reaction temperature: 25 °C).

The effect of the liquid–solid ratio on the selective leaching of tantalum and manganese was studied from 10 mL/g to 50 mL/g. The results are shown in Figure 5. The leaching efficiency of manganese increased as the liquid–solid ratio increased until the liquid–solid ratio was 40 mL/g, achieving the best leaching efficiency. Hence, 40 mL/g of liquid–solid ratio was chosen as the optimal ratio in this study.

#### 3.2.3. Effect of the Reaction Time

**Figure 6:**
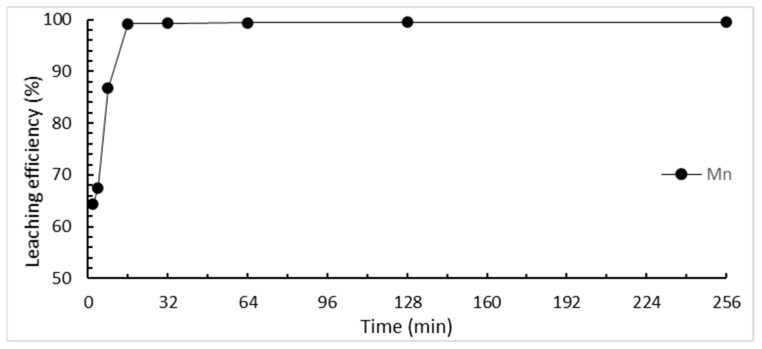
Effect of reaction time on the selective leaching efficiency. (HCl concentration: 3 M; L/S ratio: 40 mL/g; reaction temperature: 25 °C).

The effect of the reaction time on the selective leaching of tantalum and manganese was studied from 2 min to 256 min. The results are shown in Figure 6. The leaching efficiency of manganese continues to increase over time until the maximum efficiency is reached in 32 min. At the same time, we wanted to ensure that the tantalum did not dissolve. Therefore, 32 min was chosen as the optimal time in this study.

#### 3.2.4. Manganese Chemical Precipitation

**Figure 7:**
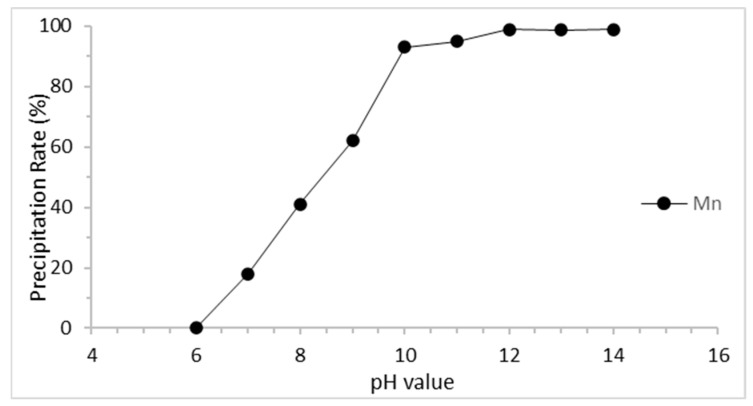
Precipitation rate of manganese.

The precipitation of manganese was investigated using the liquors obtained at the best optimal conditions in this study. Sodium hydroxide (NaOH) was used to adjust the pH value. The changes in the manganese precipitation as a function of pH value are shown in Figure 7. The manganese ions begin to precipitate when the pH value rises. At pH = 12, manganese ions almost convert to Mn(OH)_2_. Hence, pH = 12 was chosen as the best parameter for precipitation. After precipitation, Mn(OH)_2_ was analyzed by the ICP-OES (Varian, Palo Alto, CA, USA) to confirm the compositions. Table 5 shows that the purity of Mn achieves 98.5%. Due to the addition of NaOH to adjust the pH value, the productions contain a small amount of Na. Compared to previous studies [33,34,35,36,37,38], the results in this study achieved a higher leaching rate and shorter reaction times but used more acid dosage. Thus, the addition of oxidants to decrease the acid dosage and studying the temperature parameter will be discussed in future work.

### 3.3. Chlorination of Tantalum

After pretreatment and selective leaching, the epoxy resin, wires, and manganese cathodes were removed. Then, chlorination was used to extract the selective leaching residues. After the selective leaching, the remaining residues contain tantalum, graphite, and a small amount of silver chloride (AgCl). According to the previous study, tantalum could selectively react with ferrous chloride (FeCl_2_), and the reaction equation between tantalum and FeCl_2_ is as follows [31]:

2Ta (s) + 5FeCl_2_ (g) → 2TaCl_5_ (g) + 5Fe (T > 380 °C), (1)

The boiling temperature of TaCl_5_ and AgCl at standard atmospheric pressure is 234 °C and 1547 °C, respectively. When the tube furnace reaches the reaction temperature, the generated TaCl_5_ evaporates into the gas phase while the remaining compounds do not. Next, the gas of TaCl_5_ enters the condensing equipment to condense into the solid. In this study, the mixture of Ta residues and FeCl_2_ were heated at 450 °C for 3 h. The products were dissolved with hydrofluoric acid and analyzed by ICP-OES and the results are shown in Table 6. The recovery of tantalum reaches 94% and the purity of TaCl_5_ is about 99%. Comparing to previous studies [15,17,30,31,32], the results in this study achieved equivalent recovery of Ta in the chlorination process but lower than the hydrometallurgy. Therefore, the detailed reaction mechanism and optimal chlorination process will be discussed in future work.

## 4. Conclusions

As the demand for tantalum continuously increases, natural resources are mined and decrease gradually. Finding alternatives to mitigate this situation has become critical. The EcSETCs used in this study contain over 20 wt.% of tantalum and 8% of manganese, which have a great potential to provide a tremendous second resource. In this study, the epoxy resins must be removed from the EcSETCs by pyrolysis at 600 °C for 10 min, grinding, and washing. Next, the separation of iron and nickel through a magnetic separation is conducted to affect the efficiency of selective leaching. In summary, 3M of hydrochloric acid and the liquid–solid ratio of 40 mL/g at 25 °C for 32 min are chosen as the optimal parameters for selective leaching. With the optimal conditions, the leaching efficiency of Mn can achieve 99%. As Ta does not dissolve in hydrochloric acid, the leaching efficiency of Ta is almost 0%. It means that selective leaching is successful and feasible. After the filtering process, the leaching solution is divided into clear Mn ion-rich solution and Ta residues. By adjusting the pH value to 12, Mn(OH)_2_ will precipitate as one of the final productions. Then, Ta residues are washed and dried. Additionally, 48 wt.% of Ta residues and 52 wt.% of FeCl_2_ are mixed evenly and heated at 450 °C for 3 h by tube furnace. The gas of TaCl_5_ will generate and condense into the solid as the final production. In this study, the purity of Mn(OH)_2_ and TaCl_5_ is 98.5% and 99%, respectively; the recovery of Mn and Ta is 98% and 94%, respectively.

## Figures and Tables

**Table 1:** The mass ratio of each physical component in the EcSETCs (wt.%).

Electrode	Epoxy Resin	Wire
25.67%	53.41%	17.92%

**Table 2:** The concentration of the main element compositions in the EcSETCs (wt.%).

Element	Ta	Mn	Ni	Fe	Si	Ag
wt.%	20–22%	8%	10%	8%	4–6%	1%

**Table 3:** Removed efficiency of silicon, iron, and nickel after the pretreatment.

	Si	Fe	Ni	Ta
Before	46.35 mg/g	80.30 mg/g	90.75 mg/g	208.60 mg/g
After	0.01 mg/g	0.40 mg/g	0.50 mg/g	208.60 mg/g
Removed efficiency	99.8%	99.5%	99.4%	0%

**Table 4:** The leaching efficiency of manganese and tantalum with three kinds of inorganic acids.

Acid Type	Leaching Efficiency of Mn	Leaching Efficiency of Ta
HCl	99.7%	0.10%
HNO_3_	91.4%	0.13%
H_2_SO_4_	83.1%	0.01%

(Concentration: 5 mol/L; L/S ratio: 50 mL/g; reaction time: 3 h; reaction temperature: 25 °C).

**Table 5:** The metals compounds of Mn(OH)_2_ after precipitation.

Element	Mn	Na	Fe	Ni	Ta
Content	98.5%	0.7%	0.4%	0.3%	0.1%

**Table 6:** The metals compounds of TaCl_5_.

Element	Ta	Mn	Fe	Ni
Content	99%	0.2%	0.5%	0.3%
